# Generic quality of life in persons with hearing loss: a systematic literature review

**DOI:** 10.1186/s12901-018-0051-6

**Published:** 2018-01-22

**Authors:** Øyvind Nordvik, Peder O. Laugen Heggdal, Jonas Brännström, Flemming Vassbotn, Anne Kari Aarstad, Hans Jørgen Aarstad

**Affiliations:** 1 Department of Otolaryngology/Head and Neck Surgery, Haukeland University Hospital, Bergen, Norway; 2 Department of Clinical Medicine, Faculty of Medicine and Dentistry, University of Bergen, Bergen, Norway; 3 Faculty of Health and Social Sciences, Bergen University College, Bergen, Norway; 4 Department of Clinical Science, Section of Logopedics, Phoniatrics and Audiology, Lund University, Lund, Sweden; 5 Department of Health Science, Faculty of Health Sciences, University of Stavanger, Stavanger, Norway

**Keywords:** Quality of life, Hearing loss, Impairment, Distress, Depression, Anxiety, Hearing aid

## Abstract

**Background:**

To the best of our knowledge, no empirically based consensus has been reached as to if, and to what extent, persons with hearing loss (HL) have reduced generic Quality of life (QoL). There seems to be limited knowledge regarding to what extent a hearing aid (HA) would improve QoL. The main aim of the present study was to review studies about the relationship between HL and QoL. A supporting aim was to study the association between distress and HL.

**Methods:**

Literature databases (Cinahl, Pub Med and Web of Science) were searched to identify relevant journal articles published in the period from January 2000 to March 17, 2016. We performed a primary search pertaining to the relationship between HL, HA and QoL (search number one) followed by a supporting search pertaining to the relationship between distress/mood/anxiety and HL (search number two). After checking for duplications and screening the titles of the papers, we read the abstracts of the remaining papers. The most relevant papers were read thoroughly, leaving us with the journal articles that met the inclusion criteria.

**Results:**

Twenty journal articles were included in the present review: 13 were found in the primary search (HL and QoL), and seven in the supporting search (HL and distress). The literature yields equivocal findings regarding the association between generic QoL and HL. A strong association between distress and HL was shown, where distressed persons tend to have a lowered generic QoL. It is suggested that QoL is lowered among HL patients. Some studies suggest an increased generic QoL following the use of HA, especially during the first few months after initiation of treatment. Other studies suggest that HA use is one of several possible factors that contribute to improve generic QoL.

**Conclusions:**

The majority of the studies suggest that HL is associated with reduced generic QoL. Using hearing aids seem to improve general QoL at follow-up within the first year. HL is a risk factor for distress. Further research is needed to explore the relationship between HL and generic QoL, in addition to the importance of influencing variables on this relationship.

**Electronic supplementary material:**

The online version of this article (10.1186/s12901-018-0051-6) contains supplementary material, which is available to authorized users.

## Background

In 2012, the World Health Organization (WHO) estimated that 360 million people, i.e. 5.3% of the world’s population, were living with disabling hearing loss (HL), while around 15% of the world’s adult population had some degree of HL [1]. Furthermore, sensory diseases have been estimated to be the world’s second most common group of chronic disability when measured by years lived with disability [2]. HL increases with age, mostly because of age-related HL, generally referred to as presbyacusis. This term represents the sum of the environmental, sensory, metabolic and neural causes that to various extents are suggested to contribute to age-related physiological hearing loss [3, 4]. Presbyacusis cause reduced speech understanding in noisy environments, declined processing of acoustic information and impaired localization of sound sources [4]. Hearing loss is present in nearly two thirds of adults aged 70 years and older in the U.S. population [5]. Even though most people with HL suffer from presbyacusis, other factors such as other ear diseases [6], occupational noise exposure [7] and specific genetic diseases [8] may cause HL. Thus, HL may affect people at all ages and stages in life [9].

HL is often characterized by at which sound pressure level pure tones can be detected employing standard audiometric tests [3]. Presbyacusis typically causes a symmetric bilateral high frequency hearing loss. As human speech is related to relatively high frequencies, even a limited hearing loss at high frequencies may cause impaired speech intelligibility [10]. HL is often not curable, but hearing aids (HA) and other individual sound amplification devices (ISADs) may improve hearing function [11].

Patient reported outcome measures (PROMs), such as Quality of life (QoL) questionnaires, should ideally be systematically implemented in health care practices [12] as there seems to be a need for a more “holistic” approach within a modern view of health care. This calls for the inclusion of both disease-specific and generic QoL outcome measures [13]. QoL measures constitute important outcome- and state measures [14, 15], as well as an area of focus for research in its own right [14, 15]. However, there is no universally accepted definition for the concept of QoL [16, 17]. Even so, we all have a notion about what QoL is, and most people seem to have an intuitive understanding of their own QoL by referring to their own perception [16]. Thus, the concept QoL will hold different contents among different people [16].

WHO defines QoL as “An individual’s perception of their position on life in the context of the culture and value systems in which they live and in relation to their goals, expectations, standards and concerns.” This is a broad-ranging concept related to a person’s physical health, psychological state, level of independence, social relationships, personal beliefs and their relationship to salient features of their own environment. The WHO QoL definition is closely related to the WHO’s definition of health from 1948, which describes health as “physical, mental and social well-being, and not merely the absence of disease or infirmity” [16]. This is also a wide definition, in which in addition to a physical dimension, the WHO also includes well- being, environmental and psychological factors as part of health. Hence, both generic and disease-specific QoL become relevant as to disease and health [18].

Many different questionnaires have been developed with the intent of directly measuring the functional consequences of a disease; these may be termed “disease-specific” QoL questionnaires. Thus, QoL instruments intended to study the specific consequences of HL may be considered examples of such instruments [19]. The effect of HL on hearing function can usually be measured by hearing-specific questionnaires [20]*,* but to what extent HL affects generic QoL is not well agreed upon and constitutes the main aim of this study.

The most commonly used generic QoL questionnaire is the SF- 36, with more than 13,000 “hits” on Pubmed as of 2016. The SF-36 measures functional status and wellbeing [21]. This questionnaire was first used in a provisional edition in 1988 and in a standard form in 1990 [22]. Shortened questionnaires have been developed from this original, i.e. the 12-item questionnaire SF-12 [23]. Another commonly used generic questionnaire is the Euro-QoL instrument (EQ-5D). This is a standardized questionnaire intended to measure generic QoL [24], and it may be utilized within a wide range of health conditions. The EQ-5D describes five dimensions: mobility, self-care, usual activities, pain/discomfort and anxiety/depression. An index value is calculated for each individual, ranging from 1, which indicates no problems in all five dimensions, to 15, which indicate severe problems in all five dimensions. Other generic questionnaires that may be used are the Health Utility Index (HUI) and the Sickness Impact Profile (SIP) [25, 26]. General parts of disease-related questionnaires, such as the European Organization for the Research and Treatment of Cancer (EORTC) Quality of Life Questionnaire (QLQ) may also be considered generic QoL instruments [27]. Disease specific questionnaires may also include some questions about generic QoL. However, generic QoL instruments measure many aspects of QoL, and are often intended for use over a wide range of diseases. Such questionnaires are often also applicable to healthy people. Thus, generic QoL questionnaires allow comparing QoL between patient groups, as well as to data from general populations [16, 28]. The specific main aim of the present study is to review the existing literature on generic QoL obtained by generic instruments among hearing-impaired patients.

In order to assess generic QoL within a disease context, important modulating factors known to contribute to QoL may be assessed alongside the QoL measure. This may include psychosocial factors [29], personality [30, 31] and factors related to activities of daily living [32]. To study potential modulating conditions in the relationship between HL and QoL has therefore been a supporting aim when reviewing the literature in the present study.

QoL as a construct seems to be closely associated with distress, anxiety, and mood, when measured primarily in generic, but also to some extent in disease-specific QoL questionnaires [20, 33–35]. Hence, it should be of interest to study the impact of HL on distress, mood and depression. Anxiety and depression can be defined using standardized classification manuals such as the ICD-10 [36] or DSM-5 [37], while distress seems to have no such clear and universal definition. However, one may understand psychological distress as a unique discomforting, emotional state experienced by an individual that results in harm to the person, either temporarily or permanently [38]. In psychological research, distress is often quantified as the sum of anxiety and lowered mood [39]. Distress may also be utilized as an indicator of mental disease [39]. Thus, as QoL, distress, mood and anxiety are closely related concepts [40], we have conducted a search for the major publications on associations between HL and distress, anxiety and mood in order to present a more complete picture of the associations between HL and generic QoL.

### Aim of this paper

So far, no empirically based consensus about if, and in case to what extent, HL patients have reduced generic QoL has been reached. The main aim of this study was to review studies on the relationship between HL and generic QoL published in the period 2000 to present day. As a supporting aim we have also determined noted psychological explaining factors reported in the above-identified publications. As an additional investigational tool, we have reviewed papers from the same period that study HL and distress, anxiety and mood. This was done because level of distress, anxiety and mood seems closely associated to generic QoL.

## Method

### Design

Data were collected using a systematized literature review design. We performed two separate searches for relevant papers. Search number one targeted HL, HA and QoL, whereas search number two targeted HL and distress, anxiety and depression. The Prisma 2009 checklist [41] was applied during the process of writing this paper, and is available as Additional file 1.

### Searches

We suggest that literature produced over the past 15–16 years would contain most of the significant findings and results from prior studies [42]. Based on this, we set the time frame from the year 2000 up to the search date to obtain relevant literature. Moreover, we only included studies based on empirical data with an available abstract. To help narrow down the two searches in order to meet the specific aims of this study, we excluded studies concerning the hearing impaired peers or family or other caregivers. Other exclusion criteria were studies on deafness, persons with cochlea implants, dual or multi-sensorial loss, tinnitus, stigma and HL, assistive listening devices, bone-anchored hearing aids, HL and psychiatric disease, HA usage, sudden sensorineural HL, conductive HL and surgical interventions on HL. We also excluded qualitative studies as well as studies on psychiatric diseases and depression or anxiety prior to the HL.

### Search number one - HL, HA and QoL

In the primary search, we included peer reviewed original papers in English published in the period from January 2000 to March 17, 2016 (search date). Studies on QoL or health-related QoL in adult persons with sensorineural hearing loss or presbyacusis were included.

To identify relevant studies, we performed a search in the databases Cinahl, Pub Med and Web of Science. We used combinations (AND) of the following keywords:

1. Hearing disorders OR deafness OR hearing loss/partial + OR hearing loss/sensorineural + OR Tinnitus AND hearing aid OR Hearing aid fitting AND hearing loss OR hard of hearing OR loss of hearing OR hearing impair* OR hearing disorder* OR deaf* OR hearing aid* OR hearing assistive technology.
2. *Quality of life + OR Quality of Life OR health-related Quality of life OR HRQoL OR qol.*

**Fig. 1:**
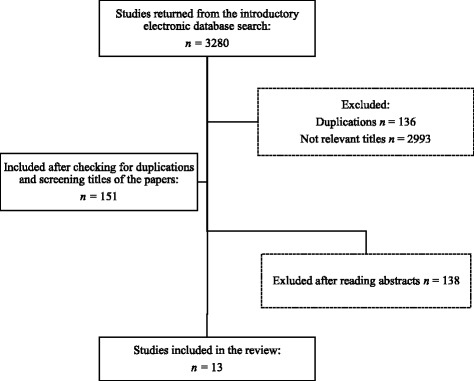
Flow chart for search number one. This flow chart shows the inclusion process following the primary search

A total of 3280 papers were found in the introductory search. After checking for duplications and screening the titles of the papers, 151 papers remained; Cinahl (*n* = 17), Pub Med (*n* = 43) and Web of Science (*n* = 91). After reading the abstracts, the remaining 35 papers were retained and thoroughly read. This left us with 13 journal articles that met the inclusion criteria (Fig. 1).

### Search number two - HL and distress, anxiety and depression

From the supporting search we included peer-reviewed original papers in English published in the period from January 2000 to October 26, 2016 (search date). This search was aimed at studies on distress, depression and/or anxiety caused by the hearing impairment, in adults with sensorineural HL.

To identify relevant studies, we performed a search on October 26, 2016, using the databases Cinahl, Pub Med and the Web of Science.

**Fig. 2:**
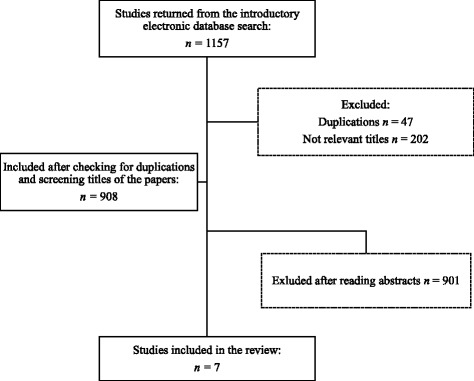
Flow chart for search number two. This flow chart shows the inclusion process following search number two

A total of 1157 papers were found in the introductory search: Cinahl (*n* = 238), Pub Med (*n* = 325), Web of Science (*n* = 594). After checking for duplications, 908 papers remained. Screening the titles of the papers, reading abstracts and then thoroughly reading the most relevant papers left us with seven journal articles to be included in this review (Fig. 2).

### Quality according to the Crowe critical appraisal tool (CCAT)

To assess the quality of the papers that met the inclusion criteria and thus were included in this review, we used the Crowe Critical appraisal tool (CCAT). The tool consists of a CCAT form and a CCAT user guide [43]. The CCAT form consists of nine category items. The first eight categories are scored from 0 to 5. The 9^th^ item states the total sum score calculated from scores at categories 1 to 8. Thus, sum scores may range from 0 to 40 points. By using this tool, we had the opportunity to systematically assess the quality of the included papers. The sum score of the CCAT for each study is presented in Tables 1 and 2.

**Table 1:** Included studies from the primary search

Study	Type of study	QoL Questionnaire used in study	First time/ experienced users?	Number of participants in study	Age	Unilateral or Bilateral HL	Range and character - HL	HA fitting	Results	CCAT score
Capoani Garcia Mondelli, M. F. and P. J. Soalheiro de Souza, 2012 [46]	Cross sectional/Longitudinal	Generic WHOQOL - bref	First time	30 (57% male)	Range: 60–90 years, mean age 76.8 years	bilateral	Moderate hearing loss. No further definition.	Before HA fitting (ISAD) and after 3 months.	Using HA (ISAD) improved the overall QoL	25
Chew, H. S. and S. Yeak, 2010 [49]	Cross sectional	Generic: SF 36	First time	80 (41% male)	Range: 50 years and over. Median age 69 years	bilateral	>25 dB PTA in the better ear.	Not specified	SF-36 lacked specificity and sensitivity in assesing the impact on HL on QoL	21
Chia, E.-M., et al., 2007 [50]	Cross sectional	Generic: SF 36	Not specified	2431	Mean age: 67 years	Unilateral and bilateral	Unilateral HI defined as HI in one ear and no HI in the other ear. Bilateral HI defined as HI in both ears. HI defined as >25 dB PTA	Not specified	Unilateral HL: No significant difference in QoLthan those whitout HL. Bilateral HL: Poorer QoL than those whitout HL.	27
Dalton, D. S., et al., 2003 [44]	5- year follow-up Longitudinal	SF-36 (Generic)	Not specified	2688, (42% male)	53–97 years, mean age 69 years	Not specified	Mild: 26–440 dB PTA HL in eighterear. Moderate to severe: >40 dB PTA in eighter ear	Not specified	HL was associated with reduced QoL.	36
Espmark, A. K. K., et al., 2002 [47]	Cross sectional	HMS (26 questions, where 4 of 20 items where related to QoL)	First time	154 (38% male)	Born 1920 or earlier	Not specified	Three groups: Normal to slight HL: <30 dB PTA. Mild HL: 30–39 dB PTA. Moderate to severe HL: ≥ 40 dB PTA	Not specified	HL was significantly associated with reduced QoL in all four dimensions in females and in two of four in males.	27
Hallberg, L. R., et al., 2008 [51]	Cross sectional	PGWB	Mixed	79 (39% male)	48–92 years, mean age 68.7 years	Bilateral	PTA low at Freq.0.5, 1 and 2 kHz was 39.6 dB. PTA high at Freq. 2,3,4 and 6 kHz was 55.5 dB	Not specified	HL was significantly associated with reduced QoL. Psychsocial consequenses of HI, such as lowered QoL, cannotbe predicted from audiometric data alone.	33
Helvik, A. S., et al., 2006 [52]	Cross sectional	PGWB	Mixed, mean duration of the HI was 15.1 years	343 (55% male)	21–94 years, mean age 69 years	Not specified	Mean threshold of hearing for the total sample was 43.0 dB	Not specified	Psychological well-being was associated with activity limitation and participation restriction, but not with the degree of HL and use of communication strategies	28
Lotfi, Y., et al., 2009 [48]	Cross sectional/Longitudinal	HHIE	First time users	207 (71% male)	˃60 years, meanage 73.01 years	Not specified	Moderate HL: 56–70 dB Profound HL: 71–90 dB	Before HA fitting and after 3 months	Significant improvement in QoL after HA fitting	19
Meyer, J. M. and S. Kashubeck-West, 2013 [55]	Cross sectional	HHIA and The meassureof psychological well-being (generic)	Not specified	277 (25% male)	18–65 years Mean age49 years	Not specified	Not specified	Not specified	Relationship between perceived severity and perceived disability acted as direct predictors to well-being and as a indirect predictors through their relationship with coping. No significant association between QoL and HL	30
Miyakita, T., et al., 2002 [54]	Cross sectional	Generic, LISZ, 13 questions about QoL	Not specified	210 retired workers, gender not specified	56–65 years, mean age 60.6 years	Not specified	Not specified	Not specified	Hearing disabillities was associated with deteriorationin QoL. No significant association between QoL and HL	23
Niemensivu, R., et al., 2015 [45]	Prospective study Including control group	Generic 15D	First time HA	949 with HI (42% male), Control group 4685 persons	Mean age: 73.8 years	Not specified	Frequencies 0.5,1,2 and 4 kHz. Four categories of HL. Mild: 25–40 dB, moderate: 41–70 dB, Severe 71–95 dB and very severe: >95 dB.	Before HA fitting (in the better ear) and after six monthts	Significant improvementin QoLafter unilateral HA fitting	29
Stark, P. and L. Hickson, 2004 [53]	Cross sectional/Longitudinal	Generic SF- 36	First time HA	131(67% male)	47–90 years, mean age 71.7 years	Not specified	Not devided in groups. PTA at0.5, 1 and 2 kHz in the better ear.	Before HA fitting and after 3 months	No significant improvements in HRQoL after HA fitting.	30
							25 dB or less: *n* = 18			
							26–35 dB:*n* = 44			
							36–46 dB:*n* = 23			
							46–55 dB: *n* = 8			
Vuorialho, A., et al., 2006 [56]	Cross sectional/Longitudinal	Generic EQ-5D in combination with HHIE-S	First time HA	98 (50% male)	61–87 years (median 77 years)	Not specified	Not specified	Before HA fitting and after 6 months	No significant QoL improvement after HA- fitting	30

*EQ-5D* EuroQol Group- 5 Dimensions

*SF- 36* Medical Outcome Study (MOS) Short Form- 36 Health Survey Scale

*15D* 15 Dimension (a standardized self-administered measure of Health related Quality of Life)

*LISZ* Life Satisfaction Index, version Z

*HMS* Hearing Measurement Scale

*PGWB* Psychological General Well Being index

*WHOQOL – bref* Abbreviated version of the WHO QoL- 100 Quality of Life assessment

*HHIE/HHIA* Hearing Handicap Inventory for the Elderly/Adults

*HHI-S* HHIE - Screening version

**Table 2:** Studies included from search number two

Authors	Type of study	Hearing loss and Distress OR anxiety OR depression	Sample size and gender	Age	Results	CCAT score
Gopinath, B., et al. (2012) [62]	Survey	Distress	811 (control group = 687) No data on gender	≥ 55 years	Older patients with HL are significantly more likely to experience emotional distress directly due to their HL.	31
Nachtegaal, J., et al. (2009) [61]	Cross- sectional	Distress, depression	1511 No data on gender	18–70 years. Divided into 5 age strata(18–29, 30–39,40–49, 50–59 and 60–70 years)	HL is negatively associated with higher distress, depression, somatization and lonliness in young and middle- aged groups.	33
Tseng, C. C., et al. (2016) [58]	Longitudinal	Depression	1717 (control group = 6868) 55% male	39–63 years. Median = 51 years	Patients with suddensensorineural hearing loss (SSHNL) are 2.17 times more at risk for depressive disorders, compared to those without SSNHL. Especially in age groups ˂ 60 years.	29
Li et al. (2014)	Survey	Depression	18,318 Male = 48%	Adults 18 years or older.	HL is significantly associated with depression, particulary in women and those younger than 70 years.	25
				18–44 years: 49.4%		
				45–69 years: 39.1%		
				≥ 70years: 11.5%		
Kramer, S. E., et al. (2002) [63]	Longitudinal (part of the LASA- study)	Depression and other chronic diseases	1506 (in the LASA- study)	55–85 years	Elderly with HL report significantly more depressive symptoms, in addition to negative association to other psychosocial variables.	20
Cetin,B., et al. (2010) [60]	Prospective	Depression and anxiety	90 (contol group = 90). All participants were male, military personel	21–30 years Mean age = 21.72 years	Higher level of depression and anxiety in the patient group, compared to the control group in the study. The duration of the HL was positevely correlated with anxiety and depression.	20
Carlsson, P.-I., et al. (2015) [24]	Retrospective	Depression and anxiety	1247 mean age = 67 years. Male = 51%	19–101 years, mean age 68 years	This study indicate greater levels of anxiety and depression among patients with severe or profound HL, than in the general population.	32

## Results

### HL and generic QoL

The range of HL was presented differently in the included studies. Five studies presented HL in groups from mild to severe HL [44–48] and five presented the number of participants over different hearing range groups [49–53]. Three studies gave no information on this [54–56]. Still, it seems that in most of the included studies, the lower limit of hearing loss was defined by a mean hearing loss exceeding 25 dB HL in the better ear at the octave frequencies from 0.5 to 4 kHz [57] (Table 1).

The included studies have used self-report questionnaires concerning QoL in adult persons with HL. The number of participants varied from 30 to 2688 (Table 1). Of the 13 studies included, 11 studies were cross-sectional, one was longitudinal [44] and one was prospective [45]. Seven studies used a generic QoL questionnaire [45, 46, 49–52, 54]. Two used a disease-specific QoL questionnaire only [47, 48], while the remaining four studies used a combination of generic and disease-specific questionnaires (Table 1). Four studies used the SF-36 in order to measure generic QoL, of which three employed the SF-36 alone [44, 49, 50]. One study combined SF-36 and a disease-specific questionnaire, the Hearing Handicap Inventory for Elderly (HHIE) [53].

In general, two of the included papers concluded that HL is substantially associated with a reduced QoL [44, 54], whereas six claimed there is a weak correlation [47, 50–53, 56] and five no [45, 46, 48, 49, 55] significant correlation between HL and generic QoL.

One study investigated both unilateral and bilateral hearing loss (HL) [50], three studies reported bilateral HL only [46, 49, 51] while the remaining nine studies provided no information on this matter. In the study that reported both unilateral and bilateral HL, persons with unilateral HL did not report significantly lower generic QoL than persons without HL. In one study, worse hearing at the high frequencies in male patients than in female patients was reported [51]. Despite this, the males had significantly better scores on generic QoL compared to the females. Furthermore, non-verbal behavior that alleviates the consequences of HL on generic QoL, such as pretending to hear, guessing what was said and avoiding interactions, was reported less used by men than by women [51].

In one study, the disease-specific questionnaire (HHIE) and the SF-36 questionnaire were employed [49]. These authors suggests that the SF-36 form lacks sensitivity and specificity in assessing the impact of HL on QoL, and suggests that untreated HL results in a significant decline in QoL, as measured with the HHIE questionnaire.

A study based on a relatively small population of 30 individuals, suggested that Individual Sound Amplification Devices (ISADs) improved the overall QoL of the individuals assessed [50]. At the same time, poor social relationships and coping skills were risk factors for reduced QoL. The study suggested that HL is one of several reasons why the elderly have depression, anxiety or other noxious emotions.

The authors of a study that investigated the effect of age at HL onset suggested that late onset HL seem to be negatively correlated to QoL [24]. That is, people who are born with HL or acquire HL in younger years seem to adapt to their HL better, without the HL affecting their QoL in adult life. This study also found that the education level was lower in persons with HL, as only 14% of the participants had university-level education [24].

One study found that there probably is an indirect connection between HL and lower QoL. The authors explain this with a decline in general health that may occur with increased age [50]. This is supported by a study that included subjects with an average age of 71.7 years that found that older people have more health problems in general. Moreover, this study suggests that QoL has many modulating factors, with HL being one of those factors [53]. Furthermore, this study suggests that it is important to understand the synergetic effect of present co-morbidities. This latter point is also addressed by a study that suggests that a varying perception of HL may be influenced by general life circumstances, and that one should not ignore the synergetic effect of multiple comorbidities on the generic QoL scores [49].

### HA use and generic QoL

Five studies measured QoL before the HA fitting, as well as after three [46, 48, 53] or six [45, 56] months following HA fitting. Four of these studies used generic questionnaires to measure QoL, while one used a disease-specific questionnaire [48]. There seems to be evidence that using HA alleviates HL and improves the quality of social relationships. The study conducted by Stark and Hickson [53] showed that the degree of HL, and extent of HA use, seems to be important for improved hearing-specific QoL. However, no significant improvement in generic QoL was reported in this study. The two other studies where QoL was measured after 3 months [46, 48], showed an improved QoL after using HA. In the two studies where QoL was measured after 6 months, one study reported that generic QoL measures yielded equivocal results [56], perhaps due to the sensitivity of the questionnaire being used. The other study [45] suggests a marginal improvement in generic QoL in adults with HL after using HA.

### HL and distress, anxiety and/or depression

In the included studies, self-report questionnaires concerning distress, anxiety or depression were collected from participants who were adult persons over 18 years with HL. The number of participants in the studies varied from 90 to 18,318 (Table 2). The gender distribution reported varied from 48 to 55% male participants [24, 58, 59]. One of the studies only had male participants [60] (see Table 2). Three studies [59, 61, 62] used data collected from large population surveys, in which data on the correlation of HL and anxiety, depression and/or distress were available. Two of the studies were based on data collected from a national health register [24] or a database [58]. The remaining two studies had data collected from a prospective study [60] and a longitudinal study [63]. The study conducted by Nachtegaal et al. [61] presented results on both distress and depression, whereas Gopinath et al. [62] presented results from distress. The rest of the included studies presented results on anxiety and depression [24, 58–60, 63]. In these studies, associations between HL and distress, anxiety or depression were only part of the results and conclusions about factors negatively associated with HL.

Of the two included studies on distress, one study suggested that hearing loss is associated with higher distress and present depression. For every decibel increase in signal to noise ratio (SNR), the distress score increased by 2%, while the odds for developing moderate or severe depression increased by 5% [61]. The other study suggested that older HL adult patients are significantly more likely to experience emotional distress [62].

In a study conducted by Hallberg et al. [51], the authors suggest that the psychosocial consequences of the HL cannot be predicted from audiometric data alone, but must be seen in the context of coping strategies, such as communication strategies. In one of these studies, two of the exclusion criteria were dementia and psychiatric disease [49]*,* while one study used limited psychiatric disease as an exclusion criterion [46].

In general, there seems to be significantly higher levels of both anxiety and depression in patients with severe or profound HL compared to a reference population. This seems to be the case even when taking into consideration that some of the patients may have developed anxiety or depression prior to the onset of HL [24]. The duration of HL seems to be positively correlated with anxiety and depression levels, thereby suggesting that the longer the amount of time with HL, the higher the levels of anxiety and depression [60]. However, many of the studies conclude that this conclusion is best supported among females and younger individuals [58, 61].

In conclusion, there seems to be a strong association between HL and depression [58, 59, 63], particularly in women and those younger than 70 years [58, 61]. Anxiety [24, 60] and distress [61, 62] also seem more prevalent among patients with HL. Thus, there is highly likely an association between distress and HL.

## Discussion

The literature included in this review yield equivocal findings regarding the association between generic QoL and HL. Some authors argue that there are strong associations [44, 54], while others find less strong [47, 50–53, 56] or no relationships at all [45, 46, 48, 49, 55]. All the included studies on associations between distress and HL give firmly support to such a conclusion, in particular concerning depression among younger individuals [58, 59, 61].

One of the two studies with the highest number of subjects, supported an association between generic QoL and HL and focused on older adults [44]. These subjects showed more severe HL the older they were. The association between increased age and severity of the HL in this study makes it difficult to conclude whether the age or the HL caused the change in generic QoL. Furthermore, when studying older adults by the use of self- reported questionnaires like a QoL questionnaire, it is important to ensure that the informants have the cognitive capacity needed to understand and complete the questionnaire. We have found no report concerning this matter in any of the published studies included in this survey. This should be a matter of future improvement of the investigational design.

Age is an example of a demographic variable that may influence generic QoL [32]. Therefore, such variables should be reported, and analyses carried out in order to estimate the relative importance of these variables. Furthermore, one should preferably adjust the QoL scores by these variables as additional analyses. This has to some extent been reported within the included papers, but no exhaustive study on this matter has been presented. Most of the included studies, however, do not lend any substantial support to the claim that demographic variables are of high importance concerning generic QoL and HL.

HL may be unilateral or bilateral. Standard procedure would be to report hearing levels from the least affected ear [64]. Nevertheless, to differentiate between the two conditions should be of importance and this was done in one investigation [50]. It should be of interest to study subjects with unilateral HL more extensively in order to acquire knowledge of any impaired QoL in this group.

Many of the studies yielding the highest CCAT-scores employed SF-36 as QoL measure, which only to some extent represents a generic HRQoL instrument. The SF-36 does not cover the full range of QoL. General symptoms are not covered [49]. More specifically health related QoL generic questionnaires could additionally be utilized in order to study whether HL affects a broader array of symptoms in persons with HL [44, 49, 53].

The associations between HL and distress, anxiety and depression are better documented than the general relationship between QoL and HL. Many factors may explain this relationship. HL may be the causative factor secondary to the social isolation caused by HL. Furthermore present comorbidity may explain both. This needs to be studied further. Distressed persons are expected to have lowered generic QoL [40]. Therefore, solely based on this association, generic QoL is suggested to be lowered among HL patients.

Regarding justifying HL treatment, improvements in both generic and disease-specific QoLs are important outcome measures, both clinically and for researchers [20]. To what extent individuals with untreated HL have lower generic QoL [49] is therefore interesting to study. A low generic QoL baseline subsequently improved after treatment constitutes an excellent HA treatment argument. A low baseline QoL among HL patients would also lend support to offering a larger range of treatments to this group beyond fitting a hearing aid [65]. The studies where generic QoL were measured following HA fitting after 3 months [46, 48, 53] or 6 months [45, 56] show equivocal findings. Some of these studies suggest increased generic QoL caused by the use of a HA, while other studies explain HA use as one of several possible factors that leads to better generic QoL. In conclusion, future generic QoL studies should be encouraged since a firm conclusion about HL and generic QoL has not yet been reached.

Despite the fact that HL may cause poorer generic QoL, and that using a HA may improve generic QoL, some studies suggest that many who are fitted with HAs, used their HA only to a limited degree [66]. This may be caused by the patients not receiving sufficient help and follow-up to master the HA [67]. Other studies on treatment show that HAs are an important contributor to increased QoL in HL patients [65]. Some studies suggest that using HAs over time seems to reverse the adverse effects of HL on QoL [62]. The process of HA fitting may also carry a placebo- effect. If so, this could also indicate that, as previously suggested [33, 68–70] concerning other diseases, generic QoL to a large extent mainly originates from the personality and thus stays more or less stable, regardless of the severity of HL.

We suggest a need for including both PROMs and physical measures in all hearing assessments [50]. Many modern HAs have the capability to log the actual use of the HAs in addition to the patient’s self-reported use. By collecting both physical and QoL data repeatedly, more robust data would be available to evaluate the strength of the relationship between the actual use of HAs and eventual improvements in QoL. By including control groups within research, one could in addition obtain more conclusive answers as to whether an improved QoL following HA fitting may be considered a Hawthorne effect [71], i.e. if the QoL improvement during HA fitting is due to the attention in this period.

For researchers, it also seems reasonable to measure additional potentially explaining variables, at several time points, when trying to determine what affects the QoL in persons with HL. Such screening would provide the opportunity to unravel why and to what extent patients with HL has lowered QoL, or even psychiatric disease. This could provide important clues on how to better help these patients. Systematic studies of HL treatment, with this perspective included, could likely provide evidence on how to better the health care services for patients with HL.

Data were collected using a literature review design with the aim to identify relevant literature published from the timespan 2000–2016 concerning patients with HL and the evaluation of their generic QoL. When using a limited time span there will always be a risk of missing important publications. This represents a possible weakness in our study that could have been overcome by extending the timespan to include previous years. Furthermore, we did not systematically search the reference list of the included papers for additional papers. This may have provided additional relevant papers and this represents a weakness in our design. Also, differences in sample sizes, age of subjects, hearing loss configurations and methodological presentations between studies complicated the comparison of results between studies.

## Conclusions

The main aim of this study was to review studies about the relationship between HL and QoL. Results of our review show that the majority of such studies suggest that HL reduces QoL. Those studies that also measured QoL after fitting of HAs suggest that HA fitting to some degree improves generic QoL at follow-up within the first year. A supporting aim was to review studies on the relationship between HL and distress, anxiety and mood. Results of our review show that HL is a risk factor for distress. We suggest that systematic studies of HL treatment, with a QoL perspective included, could provide evidence on how to better the health care services for patients with HL. As a consequence of our findings we suggest a need for including both PROMs and physical measures in persons with hearing loss, both at baseline and as outcome measures. Further research is needed to explore the relationship between HL and generic QoL, as well as the importance of various influencing variables on this relationship.

## Additional file

Additional file 1: PRISMA 2009 Checklist. (DOC 62 kb)

## Abbreviations

HA: Hearing aids
HL: Hearing loss
ISAD: Individual sound amplification device
PROM: Patient reported outcome measure
QoL: Quality of life
